# Supplementary material for: Modeling glioblastoma heterogeneity as a dynamic network of cell states
Source: Mol Syst Biol. 2021 Sep 16;17(9):e10105. doi: 10.15252/msb.202010105 (PMC8444284; doi:10.15252/msb.202010105)
Supplement: Supplementary file 6 — Source Data for Figure 5 [file MSB-17-e10105-s004.zip › Figure5A_sourcedata/GSEA_3071/hallmarks_stateIV.GseaPreranked.1623742551702/HALLMARK_MYC_TARGETS_V1.html]

Details for gene set HALLMARK\_MYC\_TARGETS\_V1[GSEA]

|  || Dataset | state53071 |
| Phenotype | NoPhenotypeAvailable |
| Upregulated in class | na\_neg |
| GeneSet | HALLMARK\_MYC\_TARGETS\_V1 |
| Enrichment Score (ES) | -0.4748179 |
| Normalized Enrichment Score (NES) | -2.5654094 |
| Nominal p-value | 0.0 |
| FDR q-value | 0.0 |
| FWER p-Value | 0.0 |
Table: GSEA Results Summary

  

Fig 1: Enrichment plot: HALLMARK\_MYC\_TARGETS\_V1      
 Profile of the Running ES Score & Positions of GeneSet Members on the Rank Ordered List

  

| PROBE | GENE SYMBOL | GENE\_TITLE | RANK IN GENE LIST | RANK METRIC SCORE | RUNNING ES | CORE ENRICHMENT || 1 | NCBP2 |  |  | 665 | 0.678 | -0.1193 | No |
| 2 | SMARCC1 |  |  | 709 | 0.654 | -0.1184 | No |
| 3 | LSM7 |  |  | 1329 | 0.434 | -0.2323 | No |
| 4 | NDUFAB1 |  |  | 1628 | 0.363 | -0.2849 | No |
| 5 | HDDC2 |  |  | 1835 | 0.327 | -0.3202 | No |
| 6 | CNBP |  |  | 1889 | 0.315 | -0.3261 | No |
| 7 | UBE2E1 |  |  | 2102 | 0.283 | -0.3632 | No |
| 8 | DDX18 |  |  | 2359 | -0.254 | -0.4092 | No |
| 9 | SNRPD3 |  |  | 2401 | -0.258 | -0.4135 | No |
| 10 | PSMA2 |  |  | 2490 | -0.268 | -0.4268 | No |
| 11 | ERH |  |  | 2555 | -0.276 | -0.4353 | No |
| 12 | EIF1AX |  |  | 2584 | -0.280 | -0.4368 | No |
| 13 | SSB |  |  | 2608 | -0.284 | -0.4373 | No |
| 14 | CUL1 |  |  | 2617 | -0.285 | -0.4348 | No |
| 15 | PSMD3 |  |  | 2623 | -0.285 | -0.4318 | No |
| 16 | XRCC6 |  |  | 2696 | -0.294 | -0.4416 | No |
| 17 | NAP1L1 |  |  | 2698 | -0.294 | -0.4376 | No |
| 18 | PSMD1 |  |  | 2727 | -0.297 | -0.4389 | No |
| 19 | TRIM28 |  |  | 2737 | -0.298 | -0.4365 | No |
| 20 | CYC1 |  |  | 2742 | -0.299 | -0.4330 | No |
| 21 | RPS3 |  |  | 2812 | -0.307 | -0.4421 | No |
| 22 | PSMC4 |  |  | 2891 | -0.318 | -0.4527 | No |
| 23 | EIF4H |  |  | 2976 | -0.330 | -0.4644 | No |
| 24 | GLO1 |  |  | 2996 | -0.332 | -0.4634 | No |
| 25 | PCBP1 |  |  | 3017 | -0.335 | -0.4626 | No |
| 26 | PPM1G |  |  | 3071 | -0.341 | -0.4681 | No |
| 27 | SNRPG |  |  | 3085 | -0.343 | -0.4658 | No |
| 28 | SLC25A3 |  |  | 3087 | -0.343 | -0.4611 | No |
| 29 | PHB |  |  | 3089 | -0.343 | -0.4565 | No |
| 30 | HDGF |  |  | 3181 | -0.357 | -0.4691 | No |
| 31 | PSMD14 |  |  | 3196 | -0.359 | -0.4668 | No |
| 32 | UBE2L3 |  |  | 3222 | -0.363 | -0.4665 | No |
| 33 | MCM2 |  |  | 3240 | -0.366 | -0.4647 | No |
| 34 | PGK1 |  |  | 3257 | -0.369 | -0.4626 | No |
| 35 | LSM2 |  |  | 3321 | -0.378 | -0.4695 | Yes |
| 36 | FAM120A |  |  | 3335 | -0.380 | -0.4667 | Yes |
| 37 | RPS5 |  |  | 3337 | -0.380 | -0.4616 | Yes |
| 38 | C1QBP |  |  | 3344 | -0.381 | -0.4574 | Yes |
| 39 | ILF2 |  |  | 3347 | -0.381 | -0.4524 | Yes |
| 40 | PSMA6 |  |  | 3359 | -0.383 | -0.4492 | Yes |
| 41 | PSMB3 |  |  | 3412 | -0.392 | -0.4537 | Yes |
| 42 | AIMP2 |  |  | 3474 | -0.404 | -0.4599 | Yes |
| 43 | CCT2 |  |  | 3491 | -0.408 | -0.4572 | Yes |
| 44 | ETF1 |  |  | 3503 | -0.410 | -0.4536 | Yes |
| 45 | CDK2 |  |  | 3521 | -0.414 | -0.4511 | Yes |
| 46 | TFDP1 |  |  | 3526 | -0.415 | -0.4460 | Yes |
| 47 | RSL1D1 |  |  | 3530 | -0.415 | -0.4408 | Yes |
| 48 | NME1 |  |  | 3548 | -0.419 | -0.4382 | Yes |
| 49 | MRPL9 |  |  | 3572 | -0.423 | -0.4367 | Yes |
| 50 | RPL22 |  |  | 3581 | -0.424 | -0.4323 | Yes |
| 51 | PSMD7 |  |  | 3597 | -0.428 | -0.4292 | Yes |
| 52 | IFRD1 |  |  | 3599 | -0.429 | -0.4234 | Yes |
| 53 | HSP90AB1 |  |  | 3618 | -0.432 | -0.4208 | Yes |
| 54 | SNRPA1 |  |  | 3638 | -0.434 | -0.4184 | Yes |
| 55 | VDAC1 |  |  | 3662 | -0.437 | -0.4167 | Yes |
| 56 | PWP1 |  |  | 3682 | -0.441 | -0.4142 | Yes |
| 57 | RAD23B |  |  | 3688 | -0.442 | -0.4090 | Yes |
| 58 | PRPS2 |  |  | 3700 | -0.445 | -0.4049 | Yes |
| 59 | NOP16 |  |  | 3705 | -0.447 | -0.3994 | Yes |
| 60 | NOLC1 |  |  | 3710 | -0.448 | -0.3938 | Yes |
| 61 | RUVBL2 |  |  | 3750 | -0.456 | -0.3950 | Yes |
| 62 | PPIA |  |  | 3778 | -0.461 | -0.3938 | Yes |
| 63 | CCT7 |  |  | 3783 | -0.462 | -0.3880 | Yes |
| 64 | PABPC1 |  |  | 3792 | -0.465 | -0.3831 | Yes |
| 65 | TCP1 |  |  | 3816 | -0.469 | -0.3809 | Yes |
| 66 | FBL |  |  | 3819 | -0.469 | -0.3747 | Yes |
| 67 | HSPD1 |  |  | 3842 | -0.474 | -0.3723 | Yes |
| 68 | AP3S1 |  |  | 3855 | -0.478 | -0.3680 | Yes |
| 69 | ABCE1 |  |  | 3867 | -0.480 | -0.3633 | Yes |
| 70 | CCT3 |  |  | 3930 | -0.494 | -0.3684 | Yes |
| 71 | SRPK1 |  |  | 3944 | -0.496 | -0.3640 | Yes |
| 72 | PTGES3 |  |  | 3953 | -0.498 | -0.3585 | Yes |
| 73 | POLD2 |  |  | 3964 | -0.502 | -0.3534 | Yes |
| 74 | PSMA1 |  |  | 3967 | -0.502 | -0.3468 | Yes |
| 75 | RPS6 |  |  | 3976 | -0.505 | -0.3412 | Yes |
| 76 | EIF4A1 |  |  | 4013 | -0.516 | -0.3409 | Yes |
| 77 | MRPS18B |  |  | 4053 | -0.526 | -0.3411 | Yes |
| 78 | ACP1 |  |  | 4084 | -0.535 | -0.3394 | Yes |
| 79 | EEF1B2 |  |  | 4153 | -0.555 | -0.3448 | Yes |
| 80 | PSMA7 |  |  | 4169 | -0.560 | -0.3398 | Yes |
| 81 | COX5A |  |  | 4172 | -0.560 | -0.3324 | Yes |
| 82 | HPRT1 |  |  | 4179 | -0.562 | -0.3256 | Yes |
| 83 | BUB3 |  |  | 4249 | -0.580 | -0.3308 | Yes |
| 84 | SNRPD1 |  |  | 4254 | -0.582 | -0.3234 | Yes |
| 85 | MCM6 |  |  | 4273 | -0.590 | -0.3186 | Yes |
| 86 | RPLP0 |  |  | 4277 | -0.591 | -0.3109 | Yes |
| 87 | CAD |  |  | 4289 | -0.595 | -0.3047 | Yes |
| 88 | POLE3 |  |  | 4336 | -0.610 | -0.3050 | Yes |
| 89 | RFC4 |  |  | 4340 | -0.611 | -0.2970 | Yes |
| 90 | KPNA2 |  |  | 4361 | -0.621 | -0.2922 | Yes |
| 91 | SSBP1 |  |  | 4392 | -0.634 | -0.2891 | Yes |
| 92 | EIF3J |  |  | 4426 | -0.643 | -0.2865 | Yes |
| 93 | EIF3B |  |  | 4433 | -0.647 | -0.2785 | Yes |
| 94 | ODC1 |  |  | 4437 | -0.649 | -0.2700 | Yes |
| 95 | SRM |  |  | 4455 | -0.656 | -0.2641 | Yes |
| 96 | PA2G4 |  |  | 4497 | -0.678 | -0.2625 | Yes |
| 97 | DDX21 |  |  | 4499 | -0.678 | -0.2532 | Yes |
| 98 | XPOT |  |  | 4515 | -0.687 | -0.2464 | Yes |
| 99 | IMPDH2 |  |  | 4521 | -0.689 | -0.2377 | Yes |
| 100 | EIF3D |  |  | 4523 | -0.692 | -0.2282 | Yes |
| 101 | EIF2S1 |  |  | 4553 | -0.707 | -0.2239 | Yes |
| 102 | NOP56 |  |  | 4556 | -0.708 | -0.2143 | Yes |
| 103 | CTPS1 |  |  | 4572 | -0.718 | -0.2072 | Yes |
| 104 | LDHA |  |  | 4574 | -0.722 | -0.1972 | Yes |
| 105 | PSMB2 |  |  | 4581 | -0.727 | -0.1882 | Yes |
| 106 | CCT4 |  |  | 4613 | -0.740 | -0.1838 | Yes |
| 107 | DUT |  |  | 4642 | -0.762 | -0.1785 | Yes |
| 108 | USP1 |  |  | 4649 | -0.763 | -0.1690 | Yes |
| 109 | SERBP1 |  |  | 4659 | -0.769 | -0.1599 | Yes |
| 110 | KPNB1 |  |  | 4681 | -0.786 | -0.1529 | Yes |
| 111 | CDC45 |  |  | 4702 | -0.798 | -0.1456 | Yes |
| 112 | MCM7 |  |  | 4704 | -0.800 | -0.1346 | Yes |
| 113 | CCT5 |  |  | 4740 | -0.822 | -0.1298 | Yes |
| 114 | RANBP1 |  |  | 4741 | -0.823 | -0.1182 | Yes |
| 115 | RAN |  |  | 4743 | -0.824 | -0.1069 | Yes |
| 116 | MCM5 |  |  | 4792 | -0.853 | -0.1042 | Yes |
| 117 | CDK4 |  |  | 4810 | -0.874 | -0.0952 | Yes |
| 118 | EIF2S2 |  |  | 4853 | -0.915 | -0.0905 | Yes |
| 119 | PABPC4 |  |  | 4855 | -0.918 | -0.0778 | Yes |
| 120 | MYC |  |  | 4883 | -0.947 | -0.0697 | Yes |
| 121 | PRDX4 |  |  | 4889 | -0.954 | -0.0573 | Yes |
| 122 | MCM4 |  |  | 4947 | -1.011 | -0.0541 | Yes |
| 123 | RRM1 |  |  | 4990 | -1.074 | -0.0472 | Yes |
| 124 | MAD2L1 |  |  | 5007 | -1.105 | -0.0348 | Yes |
| 125 | NPM1 |  |  | 5048 | -1.184 | -0.0259 | Yes |
| 126 | CCNA2 |  |  | 5051 | -1.186 | -0.0096 | Yes |
| 127 | PCNA |  |  | 5060 | -1.214 | 0.0059 | Yes |
| 128 | CDC20 |  |  | 5082 | -1.249 | 0.0194 | Yes |
| 129 | TYMS |  |  | 5156 | -1.477 | 0.0260 | Yes |
Table: GSEA details [plain text format]

  

Fig 2: HALLMARK\_MYC\_TARGETS\_V1: Random ES distribution      
 Gene set null distribution of ES for **HALLMARK\_MYC\_TARGETS\_V1**
